# Double ventricular response with concomitant atrioventricular nodal reentrant tachycardia

**DOI:** 10.1016/j.hroo.2026.05.024

**Published:** 2026-06-10

**Authors:** David T. Zhang, Abhijeet Singh, Eric J. Rashba, Roger Fan

**Affiliations:** Division of Cardiology, Department of Medicine, Stony Brook Medicine, Stony Brook, New York

**Keywords:** Double ventricular response, Double fire, Dual AV nodal physiology, AV nodal reentrant tachycardia, Slow pathway ablation

**What We Learned**

▪ The double ventricular response is a rare manifestation of dual atrioventricular (AV) nodal physiology that can coexist with typical AV nodal reentrant tachycardia (AVNRT).
▪ Minimal retrograde slow pathway conduction is a key determinant enabling simultaneous antegrade fast and slow pathway conduction.
▪ Slow pathway ablation effectively eliminates both the double ventricular response and AVNRT.

## Introduction

Dual atrioventricular (AV) node physiology is usually seen in the setting of AV nodal reentrant tachycardia (AVNRT), the most common form of supraventricular tachycardia (SVT) in adults.^1^^,^^2^ Typical slow-fast AVNRT requires antegrade block in the fast pathway of the AV node with conduction down the slow pathway, followed by retrograde conduction up the fast pathway once refractoriness has recovered.^3^ Rarely, a single atrial impulse can conduct antegrade simultaneously down both the fast and slow AV nodal pathways, producing 2 ventricular depolarizations—a phenomenon known as a “double ventricular response.”^4^, ^5^, ^6^ This phenomenon can exist as an isolated finding or as a manifestation of a sustained nonreentrant tachycardia, termed dual AV nodal nonreentrant tachycardia (DAVNNT).^7^^,^^8^ Although both double ventricular response and AVNRT share the substrate of dual AV nodal physiology, their coexistence in the same patient has rarely been reported.^1^^,^^9^ We present a case of a patient who demonstrated both a double ventricular response and inducible AVNRT during the same electrophysiology study (EPS).

## Case report

**Figure 1:**
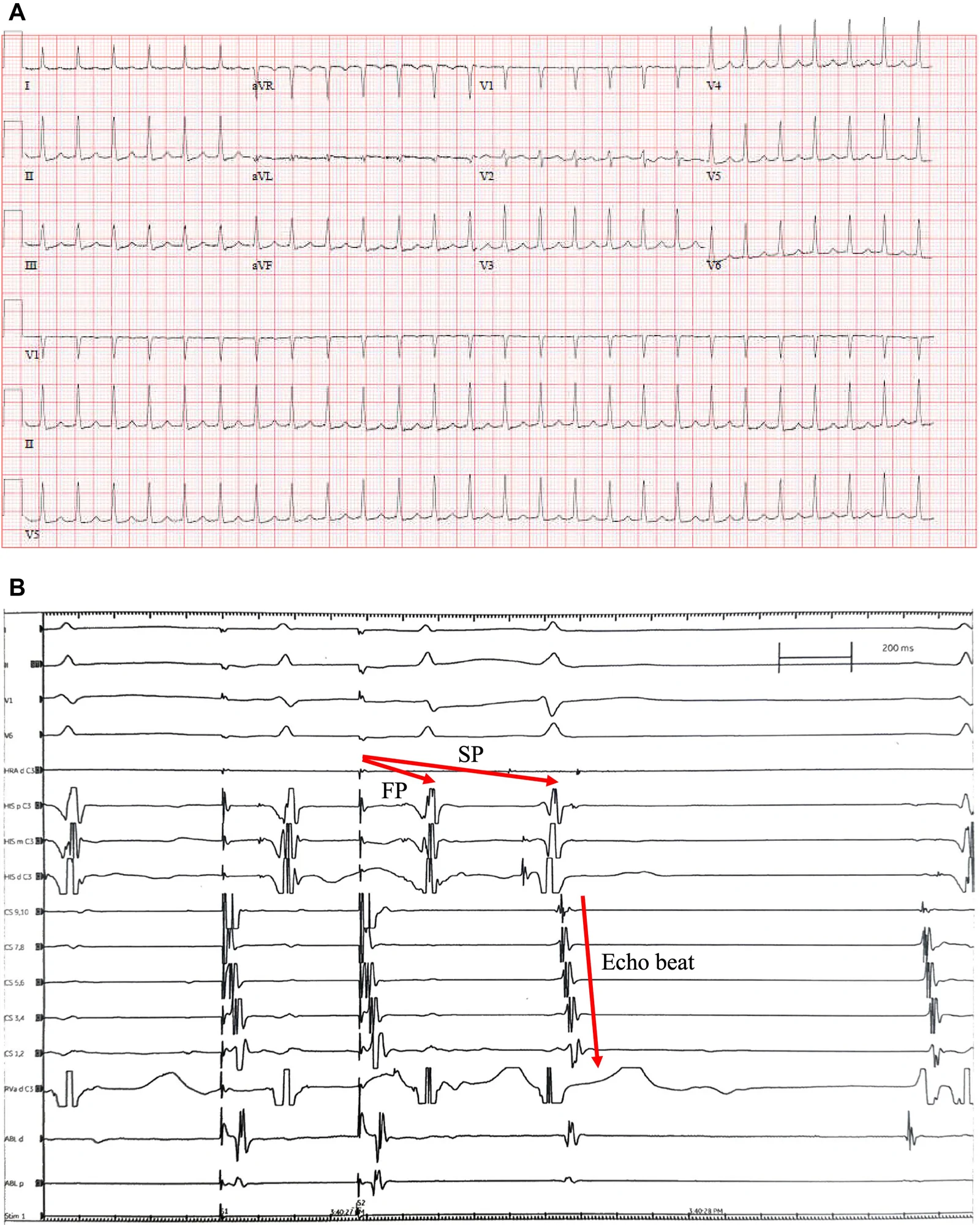
**A:** Electrocardiogram with short-RP supraventricular tachycardia, which terminated with adenosine. **B:** Atrial extrastimulus producing a double ventricular response with antegrade conduction down both the FP and SP of the atrioventricular node during an electrophysiology study for clinical atrioventricular nodal reentrant tachycardia. There is also an atrial echo beat noted on the second QRS of the double ventricular response. FP = fast pathway; SP = slow pathway.

A 64-year-old woman with obstructive sleep apnea and Sjögren’s syndrome presented on multiple occasions over several years for evaluation of palpitations. Electrocardiograms demonstrated a short-RP SVT at rates of 160–190 beats per minute (Figure 1A). All episodes terminated with adenosine administration. Given the recurrent and symptomatic nature of her arrhythmia, she consented to an EPS with SVT ablation.

The patient presented to the electrophysiology laboratory in sinus rhythm. Baseline conduction intervals, including the AH and HV intervals, were within normal limits. There was no evidence of an accessory pathway during atrial or ventricular pacing. Atrial programmed electrical stimulation at a drive cycle length of 600 ms demonstrated dual AV nodal physiology with a characteristic AH “jump.” At a critical coupling interval, a single atrial extrastimulus produced simultaneous conduction down both the fast and slow AV nodal pathways, resulting in 2 His bundle deflections and 2 QRS complexes—a double ventricular response (Figure 1B). In addition, an atrial echo beat was noted after the second QRS complex conducted over the slow pathway, reflecting retrograde conduction up the fast pathway. With the infusion of isoproterenol, a sustained short-RP tachycardia with a cycle length of 290 ms was induced. Right ventricular overdrive pacing led to SVT termination; however, the transition zone demonstrated a fully paced QRS complex before atrial electrogram advancement, ruling out AV reentry tachycardia.^10^ The tachycardia spontaneously and repetitively terminated with an atrial signal, ruling out atrial tachycardia. A diagnosis of AVNRT was made, and slow pathway modification was performed with radiofrequency energy. After ablation, there was no further inducible tachycardia or double ventricular response. The patient did well on follow-up.

## Discussion

Our patient demonstrates the rare coexistence of a double ventricular response and typical AVNRT during the same EPS. Both phenomena arise from the shared substrate of dual AV nodal physiology but manifest through distinct electrophysiological mechanisms.

The double ventricular response, first described in 1975, occurs when a single atrial impulse conducts anterogradely over both the fast and slow AV nodal pathways, generating 2 sequential His bundle depolarizations and ventricular complexes.^4^ For this to occur, 2 conditions must be met: (1) the conduction delay in the slow pathway must be sufficient to allow the distal His-Purkinje system to recover excitability after antegrade fast pathway conduction, and (2) retrograde conduction from the fast pathway impulse into the slow pathway must be absent or minimal, thereby preventing collision with the oncoming antegrade slow pathway wavefront.^5^^,^^6^^,^^11^ Our patient, at baseline during the EPS, only demonstrated a double ventricular response without inducible tachycardia. However, with the sympathomimetic effect of isoproterenol, the AV node’s conduction properties were altered sufficiently to create an excitable gap promoting reentry, leading to sustained AVNRT.

The double ventricular response with concomitant AVNRT has rarely been reported in the literature. Similar to our case, Kanjwal^12^ described a double ventricular response with an atrial echo in a patient with AVNRT. Furthermore, antegrade dual AV nodal conduction, when sustained, produces DAVNNT—a nonreentrant tachycardia frequently misdiagnosed.^7^^,^^8^ Extending the first criterion described earlier, it has been demonstrated that extreme slow pathway conduction delay (mean AH >500 ms) is a precondition for DAVNNT.^13^ Likewise, the coexistence of DAVNNT and AVNRT has been infrequently documented. 2 case reports have previously described concomitant DAVNNT and AVNRT, concluding that retrograde fast pathway conduction quality determines which arrhythmia manifests.^1^^,^^9^ Taken together, these reports highlight how rarely the conditions for both phenomena coexist in a single patient.

Our case adds to the limited literature demonstrating that dual AV nodal physiology can manifest as multiple distinct arrhythmia mechanisms within the same patient. The successful elimination of both phenomena with slow pathway ablation confirms the shared substrate underlying both the double ventricular response and AVNRT.

## Conclusion

A 64-year-old woman with recurrent short-RP SVT underwent EPS demonstrating dual AV nodal physiology with a double ventricular response and typical AVNRT. Both phenomena were treated with slow pathway ablation.

## Disclosures

The authors have no conflicts of interest to disclose.
